# Supplementary material for: The interactome of intact mitochondria by cross-linking mass spectrometry provides evidence for coexisting respiratory supercomplexes
Source: Mol Cell Proteomics. 2017 Dec 8;17(2):216–32. doi: 10.1074/mcp.RA117.000470 (PMC5795388; doi:10.1074/mcp.RA117.000470)
Supplement: Supplemental Data [file supp_RA117.000470_133922_0_supp_23978_fzffwf.zip › spectra_annotation/mito_DR_spectra_annotation/335-1-11-1-11-1.pdf]

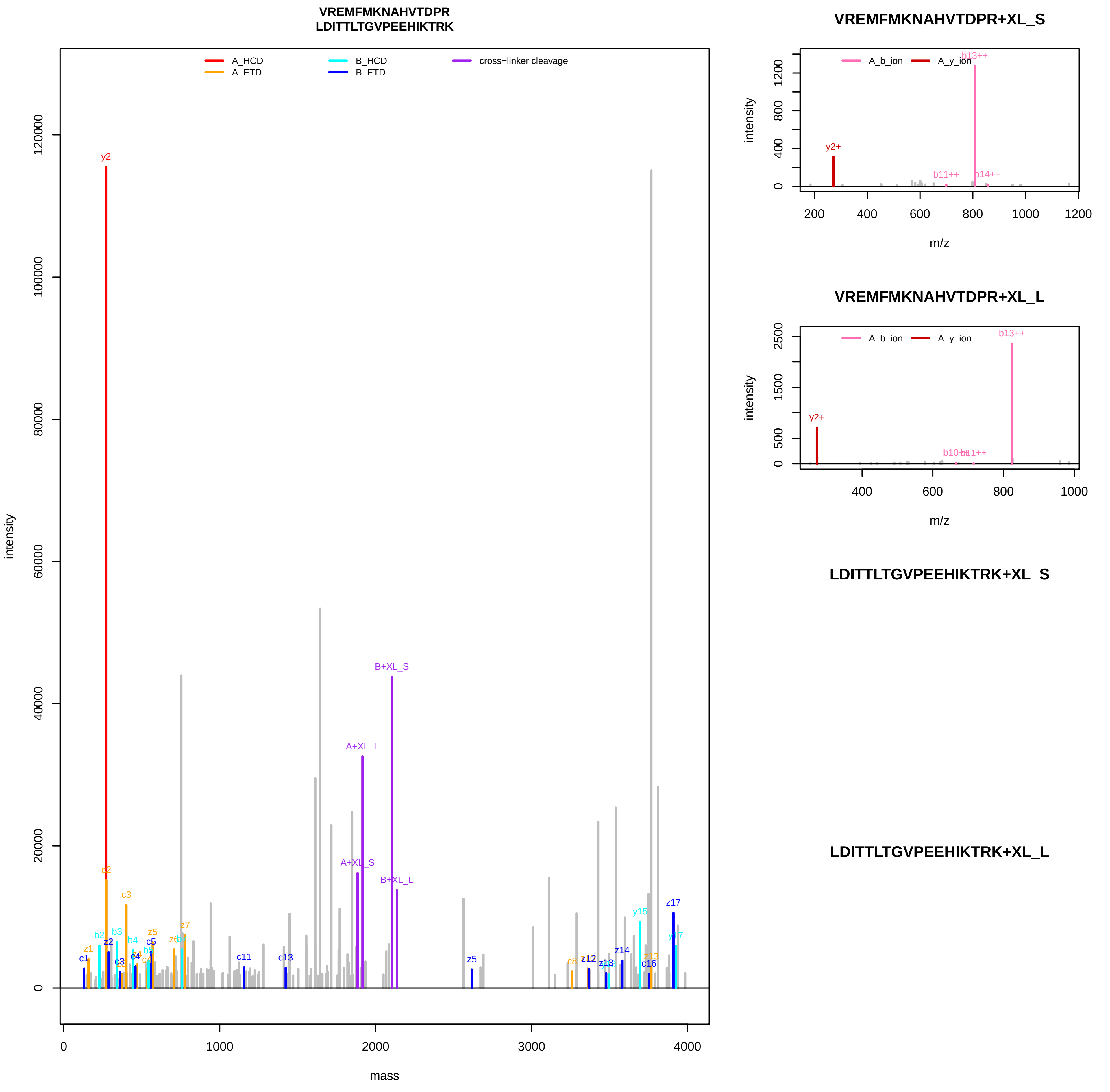
VREMFMKNAHVTDPR
LDITTLTGVPEEHIKTRK
A_HCD
A_ETD
B_HCD
B_ETD
cross−linker cleavage
intensity
mass
B+XL_S
A+XL_L
A+XL_S
B+XL_L
y2
c2
c3
c1
z1
b2
b3
z2
b4
c4
c5
z5
z7
c11
c13
z5
c8
z12
z13
z14
y15
c16
z17
y17
VREMFMKNAHVTDPR+XL_S
A_b_ion
A_y_ion
b13++
y2+
b11++
b14++
m/z
VREMFMKNAHVTDPR+XL_L
b10++
LDITTLTGVPEEHIKTRK+XL_S
LDITTLTGVPEEHIKTRK+XL_L
